# Water Insecurity, Sociopolitical Instability, and Resurgence of Cholera in Haiti, 2022: An Outbreak Investigation

**DOI:** 10.4269/ajtmh.24-0755

**Published:** 2025-07-01

**Authors:** Stanley Juin, Edwige Michel, Wilfredo R. Matias, Evenel Thermidor, Molly F. Franke, Michelle V. Evans, Hetsner Denis, Yodeline Guillaume, Roberta Bouilly, Wisnel Mathurin, Katilla Pierre, Valusnor Compere, Kenold Rendel, Hebrelienne Amelus, Lesly Andrecy, Gerard A. Joseph, Jacques Boncy, Donald Lafontant, Louise C. Ivers

**Affiliations:** ^1^ Center for Global Health, Massachusetts General Hospital, Boston, Massachusetts;; ^2^ Direction d’Épidémiologie des Laboratoires et de Recherche, Ministère de la Santé Publique et de la Population, Port-au-Prince, Haiti;; ^3^ Center for Global Health Division of Infectious Diseases, Massachusetts General Hospital, Boston, Massachusetts;; ^4^ Division of Infectious Diseases, Brigham Women’s Hospital, Boston, Massachusetts;; ^5^ Direction Nationale d’Eau Potable, Port-au-Prince, Haiti;; ^6^ Department of Global Health and Social Medicine, Harvard Medical School, Boston, Massachusetts;; ^7^ Laboratoire National de Santé Publique, Ministère de la Santé Publique et de la Population, Port-au-Prince, Haiti

## Abstract

We investigated a resurgence of cholera in Haiti in 2022, occurring after 3 years without cases. We analyzed data from the National Cholera Surveillance System for the first reported cases in 2022 and interviewed field epidemiology teams. We used logistic regression to identify risk factors associated with confirmed cholera. Few suspected cases reported ever receiving oral cholera vaccine (14%) or previous hospitalization for cholera (7%). Recently changing water sources were associated with culture-confirmed cholera (odds ratio 5.55, 95% CI 2.13–15.12). Spatial analysis of cholera cases revealed significant clustering (*P* = 0.001) and low prevalence (11%, 15 of 136) of residual chlorine in private water points in the affected area. Qualitative analysis suggested a link between cholera resurgence and an acute lack of access to safe water because of gang violence. Ongoing response to cholera in Haiti is crucial, alongside sustained investment in long-term solutions like improved water and sanitation infrastructure and addressing socioeconomic issues.

## INTRODUCTION

Cholera, an acute watery diarrhea (AWD) caused by *Vibrio cholerae*, emerged as a public health threat in Haiti in 2010. Between October 2010 and February 2019, the Haitian Ministry of Health (MSPP) reported 820,000 suspected cases and 9,792 deaths,^1^^,^^2^ although the true burden was likely higher.^3^^,^^4^ To address this epidemic, MSPP launched a 10-year elimination plan involving oral cholera vaccination (OCV), rapid response teams, and improved hygiene and water treatment practices.^5^^,^^6^ From February 2019 to September 2022, no AWD cases were confirmed as being a result of *V. cholerae*.^7^^,^^8^

However, political unrest, gang violence, and a port blockade causing food, water, and fuel shortages coincided with severe AWD cases in the Ouest Department on September 30, 2022.^9^ MSPP reported two culture-confirmed cholera cases on October 1, 2022, with genomic sequences matching the 2010 strain.^10^^,^^11^ We conducted an outbreak investigation to understand factors associated with cholera resurgence.

We used a mixed-methods approach, combining retrospective analysis of surveillance data with home visits to complete missing information. MSPP’s directorate of epidemiology, laboratory, and research (DELR) manages a case-based diarrhea reporting system as part of MSPPs national cholera surveillance system (NCSS). Health facility officers upload notification forms capturing demographic, clinical, and risk factor data. During the initial phase of the outbreak, health facilities also sent stool samples from all cholera-suspect cases to the national laboratory for culture.^12^ We reviewed NCSS cases registered between September 30 and November 17, 2022, that met MSPP’s case definition of suspected cholera (i.e., ≥3 AWD episodes within 24 hours, with or without dehydration). Active cases not registered by end-of-study activities on November 17 were not included. Demographic, clinical, and risk factor data were abstracted and trained epidemiologists conducted in-person data collection to address missing data. Data for hospitalized patients were collected at bedside; data for discharged or deceased patients were collected at their residences. GPS coordinates were recorded during home visits with verbal consent. We interviewed five of eight field team members about their observations on factors relevant for cholera transmission, including living conditions, water and sanitation access, and the overall community context.

To identify risk factors, we compared culture-confirmed cholera cases and non-cholera diarrhea cases using univariable analyses. Factors with *P*-value <0.2 were included in a multivariable logistic regression. *P*-values <0.05 were considered significant. Case GPS coordinates were mapped with water source locations from a National Directorate of Potable Water and Sanitation (DINEPA) water quality survey conducted in November 2022. We performed geospatial analysis to assess the spatial distribution of cholera cases and their association with water sources. Quantitative analyses were conducted using R V4.2.2. Qualitative interviews provided thematic insights.

The outbreak investigation was undertaken by DELR. Written consent was not required by the Haitian National Bioethics Committee.

**Figure 1.**
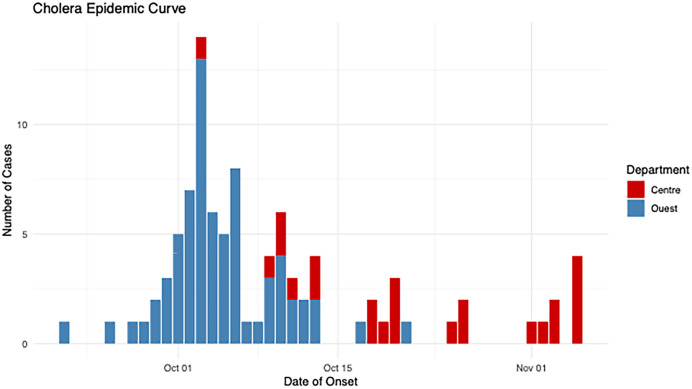
Time distribution of culture-confirmed cholera cases by date of symptom in Ouest and Centre Departments, Haiti, September–November 2022.

From September 30 to November 17, 2022, 98 cholera-suspect cases were considered (excluding seven lacking stool cultures). At investigation, 70 (71%) were discharged, 22 (22%) hospitalized, five had died, and one had missing outcome data. All cholera-suspect cases were from Ouest (73.5%) and Centre (26.5%) Departments, with 88% concentrated in Cité-Soleil (Ouest) and Mirebalais (Centre). Culture-confirmed cases had a similar distribution, but Centre’s first confirmed case developed symptoms 12 days after Ouest’s (Figure 1).

**Figure 2.**
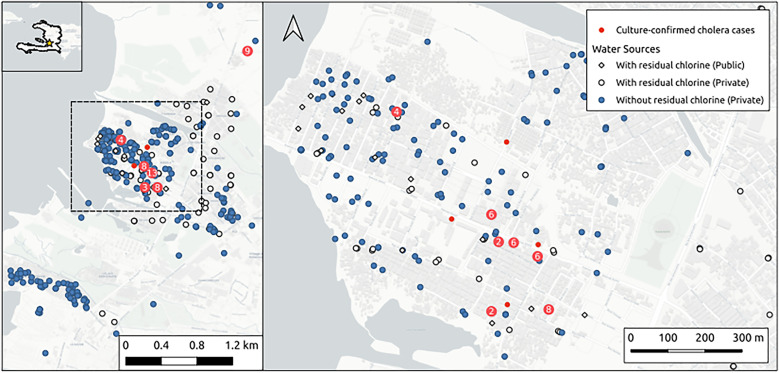
Geographic distribution of culture-confirmed cholera cases, water access, and quality in Ouest Department, Haiti, September–November 2022. These maps display the spatial distribution of culture-confirmed cholera cases (red dots) in relation to unchlorinated (blue dots), private chlorinated (circles), and public chlorinated (diamonds) water sources in an urban area of Port-au-Prince, Haiti, from September to November 2022. The left pane provides a broader view of the study area, whereas the right pane (corresponding to the demarcated dashed box in the left pane) zooms in on the majority of Cité-Soleil, where the first cases of resurgent cholera were documented. The inset map (left pane, top left) shows the location of the study area within Haiti. The scale bars indicate distances in kilometers and meters for reference. To protect individuals’ privacy, the cases are presented as micro-clusters rather than individual data points.

Spatial analyses (Figure 2) were limited to Ouest Department cases, as only these cases had home visits and GPS data. Of 60 Ouest cholera-suspect cases with valid GPS coordinates, 48 were culture-confirmed. These cases clustered significantly (Pearson’s χ^2^ = 8179.8, *P* = 0.001) based on a conditional Monte Carlo test of complete spatial randomness using quadrat count. The median pairwise distance between cases was 328.2 m (range 0.0–11,334 m). Only two cases in Ouest Department resided outside Cité-Soleil, with no clear link between them on interview. DINEPA surveys showed 160 water points in Cité-Soleil (Figure 2). Twenty-four were served by DINEPA and all (100%) had residual chlorine. A total of 136 were privately managed, with only 15 (11%) having residual chlorine. Likelihood ratio tests of Poisson process models showed no significant difference in median proximity between a case’s residence and water points with or without residual chlorine.

Risk*-*factor analyses were conducted only for cases from Ouest Department because the small sample size of cases from Centre Department precluded analysis. Table 1 compares risk factors for culture-confirmed *V. cholerae* diarrhea and non-cholera diarrhea. Most suspected cases (54%) were <10 years old. Only 13% reported ever having received OCV. In multivariable analysis, reporting a recent water source change was associated with an increased risk of cholera diarrhea (odds ratio [OR] 12.95, 95% CI 2.11–79.4, *P* = 0.006), whereas reporting prior OCV vaccination was associated with decreased risk of cholera diarrhea (OR 0.20, 95% CI 0.04–0.87, *P* = 0.032).

**Table 1:** Description of the first suspected cases of resurgent cholera in Ouest Department, Haiti, September–November 2022

Risk Factors	AWD, *N*	Culture Negative for *V. cholerae, n* (%)	Culture Positive for *V. cholerae, n* (%)	Unadjusted Odds Ratio (95% CI)	*P*-Value	Adjusted Odds Ratio (95% CI)	*P*-Value
West	72	17 (100.0)	55 (100.0)	**–**	**–**	**–**	**–**
Age group (years)							
0–4	22	5 (29.4)	17 (30.9)	Ref	**–**	**–**	**–**
5–9	15	4 (23.5)	11 (20.0)	0.8 (0.17–3.89)	0.784	**–**	**–**
10–19	18	4 (23.5)	14 (25.5)	1.02 (0.22–4.86)	0.969	**–**	**–**
20–39	8	3 (17.6)	5 (9.1)	0.49 (0.08–3.04)	0.423	**–**	**–**
40+	9	1 (5.9)	8 (14.5)	2.35 (0.30–49.0)	0.467	**–**	**–**
Gender							
Male	47	12 (70.6)	35 (63.6)	Ref	**–**	**–**	**–**
Female	22	5 (29.4)	17 (30.9)	1.37 (0.43–4.82)	0.590	**–**	**–**
Missing	3	0 (0.0)	3 (5.5)	**–**	**–**	**–**	**–**
Travel							
No	63	15 (88.2)	48 (87.3)	Ref	**–**	**–**	**–**
Yes	9	2 (11.8)	7 (12.7)	1.09 (0.23–7.86)	0.916	**–**	**–**
High-risk setting*							
No	17	3 (17.6)	14 (25.5)	Ref	**–**	**–**	**–**
Yes	54	13 (76.5)	41 (74.5)	0.67 (0.14–2.48)	0.581	**–**	**–**
Missing	1	1 (5.9)	**–**	**–**	**–**	**–**	**–**
High-risk contact^†^							
No	66	15 (88.2)	51 (92.7)	Ref	**–**	**–**	**–**
Yes	6	2 (11.8)	4 (7.3)	0.58 (0.10–4.53)	0.562	**–**	**–**
Drinking water source^‡^							
Treated water only	4	0 (0.0)	4 (7.3)	N/A^‖^	**–**	**–**	**–**
Unsafe water	68	17 (100.0)	51 (92.7)	N/A	**–**	**–**	**–**
Change source of water^§^							
No	7	5 (29.4)	2 (3.6)	Ref	**–**	**–**	**–**
Yes	65	12 (70.6)	53 (96.4)	11.4 (2.11–83.9)	0.007^¶^	12.95 (2.11–79.4)	0.006^¶^
Place of defecation							
Latrines	11	1 (5.9)	10 (18.2)	3 (1.51–5.93)	0.275	**–**	**–**
Open defecation	44	11 (64.7)	33 (60.0)	Ref	**–**	**–**	**–**
Pit	13	4 (23.5)	9 (16.4)	0.75 (0.19–2.92)	0.678	**–**	**–**
WC	4	1 (5.9)	3 (5.5)	1 (0.09–10.6)	1.000	**–**	**–**
Prior (ever) hospitalization for cholera							
No	71	16 (94.1)	55 (100.0)	N/A	**–**	**–**	**–**
Yes	1	1 (5.9)	0 (0.0)	N/A	**–**	**–**	**–**
Prior (ever) oral cholera vaccination							
No	62	12 (70.6)	50 (90.9)	Ref	**–**	**–**	**–**
Yes	10	5 (29.4)	5 (9.1)	0.24 (0.05–0.98)	0.040^#^	0.2 (0.04–0.87)	0.032^#^

AWD = acute watery diarrhea; N/A = not applicable; Ref = reference; *V*. = *Vibrio*; WC= water closet.

* High-risk setting refers to a place with high risk of person-to-person transmission and includes public markets, funerals, prisons, seaports, or hospitals.

^†^ High-risk contact refers to visiting or receiving a visit from foreigners or people who have traveled outside the department.

^‡^ Drinking water sources include treated water and untreated water (rivers, springs, wells, rainwater).

^§^ Change source of water indicates having changed the primary drinking water source in the 2 weeks prior to being sick.

^¶^ *P* < 0.01 highly significant.

^‖^ Not applicable: all variables with cells of zero observations are excluded from the univariable analysis.

^#^ *P* < 0.05 statistically significant.

Qualitative interviews with field epidemiology teams identified several themes. First, universally poor living conditions in the impacted area including overcrowded, flood-prone, single-room homes. Second, residents described a total blockade of the affected area in Ouest Department because of gang activity; this was corroborated by outbreak response teams having to negotiate access to enter certain areas for emergency response activities. Third, the field team witnessed visibly contaminated water and reported residents’ description of being unable to travel to safer water sources because of gang-related activities in the area. DINEPA could not pump water to its network in Cité-Soleil during the days preceding the outbreak because of this blockade.^9^

Our investigation revealed a rapid, highly clustered outbreak of AWD, with almost three*-*quarters of initial confirmed cholera cases concentrated in Cité-Soleil, a densely populated informal settlement in the Port-au-Prince area. Most cases (75%) were culture confirmed as *V. cholerae*. The sharp initial rise and case proximity in Cité-Soleil, amid prevalent, inadequately treated water points and recent changes in water sources, strongly suggests a common source outbreak, though we were unable to pinpoint a specific source. These initial cases were followed by a less explosive surge in Mirebalais (Centre), 56.5 km away. No epidemiological link between Cité-Soleil and Mirebalais cases was found, though we hypothesize the spread was through activities like the movement of goods and people.

Traditional cholera risk factors like unsafe water and poor sanitation were prevalent in both our quantitative and qualitative analyses. Our data showed that 94.4% of suspected cholera cases used unsafe drinking water and 61.1% practiced open defecation, underscoring the importance of basic water, sanitation, and hygiene in preventing diarrheal disease. Confirmed cholera cases were more likely to report a recent change in water source, aligning with previous findings on the risk of waterborne diseases associated with water supply interruption.^13^^,^^14^ Affected communities faced precarious conditions, with a lack of agency over access to clean water. Private water facilities did not have adequate levels of chlorination, and public water facilities were unavailable, even weeks after the start of the outbreak. DINEPA reported being unable to pump water to public water stands because of fuel shortages, leaving community members with limited water security.

As part of an escalating series of sociopolitical crises, gang violence in Haiti increased substantially throughout 2022, with armed groups controlling large parts of the capital. In the weeks prior to this cholera outbreak, a blockade and fuel shortages crippled transportation, hindering access to essential services,^15^ severely impacting daily life, leaving hospitals struggling, businesses closed, and exacerbating a humanitarian crisis.^9^ Qualitative reports from this outbreak investigation suggest an association between violence and interruption in water supply, thereby adding to the evidence on pathways between political instability, violence, and cholera, and highlighting the vulnerability of public health in conflict.^16^^,^^17^

Most of the cases were children, with over 50% of AWD cases and culture-confirmed cholera cases being under 10 years old, consistent with patterns in cholera-endemic areas.^2^ Factors likely accounting for this include low natural immunity and low oral cholera vaccine coverage. Regional cholera vaccination campaigns for those 1 year or older had not been held since 2016 (Ouest) and 2017 (Centre); therefore, children aged 6 and under at the time of this outbreak had either not been born or were ineligible for vaccination. In addition, oral cholera vaccines are known to have only modest protective effectiveness for young children.^2^^,^^18^^,^^19^ Although the self-reported OCV rate was low for the studied population (13%), on adjusted analysis, culture-confirmed cholera was less prevalent amongst those who reported ever receiving OCV.

Our study has limitations. Gang activity hindered home visits and data collection. The DINEPA water survey was conducted after the outbreak began, as part of water intervention efforts. Pre-outbreak water quality data were unavailable but likely worse than that reflected in the current survey given disruptions to DINEPA services, illustrating the gravity of the situation and the need for continued support for affected communities. Additionally, health facility-reported AWD likely underestimates the true burden of infections. Finally, a retrospective design restricts our ability to establish causal links between risk factors and cholera.

Our investigation into Haiti’s 2022 cholera resurgence revealed a concentrated outbreak in Cité-Soleil and Ouest Department among an immunologically susceptible and socially vulnerable population facing acute water insecurity and disruptions related to gang violence. Recent water source changes increased cholera risk, whereas prior OCV immunization was protective. Urgent humanitarian and long-term multisectoral interventions are needed to prevent future cholera outbreaks in Haiti.
